# The Beta Amyloid Core Hexapeptide Protects against Full-Length Beta Amyloid-Induced Alteration of Dendritic Spine Morphology and Density

**DOI:** 10.1523/ENEURO.0044-25.2025

**Published:** 2025-09-09

**Authors:** Ruth M. Shontell, David Araki, Kendra M. Ormsbee, Donovan D. Delgado, Robert A. Nichols

**Affiliations:** Department of Cell & Molecular Biology, John A. Burns School of Medicine, University of Hawai’i at Manoa, Honolulu 96813, Hawaii

**Keywords:** beta amyloid, dendritic spines, hippocampal neuronal cultures, protective peptides

## Abstract

Pathological levels of beta amyloid (Aβ) lead to disruption and elimination of synapses in brain as the result of direct neurotoxicity as well as neuroinflammation. The synaptic impact of beta amyloid includes altered morphology and reduced number of dendritic spines at excitatory synapses, evident in the brains of individuals with Alzheimer’s disease. Here, we assessed the ability of an identified neuroprotective peptide, YEVHHQ, derived from the N-terminal domain of Aβ, known as the AβCore, to protect against Aβ-induced alterations in dendritic spines. Our approach involved both 2D and 3D imaging of spine morphology in hippocampal neuron cultures from mice of either sex, with the 3D imaging focusing on the postsynaptic density (PSD), as its morphology is tightly correlated with synaptic strength, and presynaptic terminal morphology and density to assess the impact on both sides of the synapse. We present evidence for uniform prevention by the AβCore of Aβ-induced reductions in spine cross-sectional size and density as well as PSD surface area and volume. In addition, the AβCore alone increased the presynaptic terminal volume in parallel to the reversal of Aβ-induced changes in spine and PSD size. Together, these results provide support for reversal of structural changes underlying the functional reversal by the AβCore of Aβ-induced impairment of synaptic dynamics.

## Graphical abstract

**Figure:**
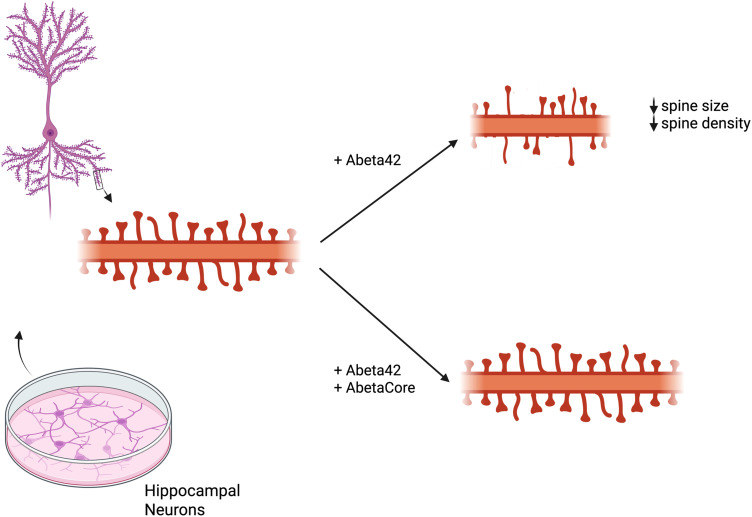
Visual Abstract

## Significance Statement

Dendritic spines are dynamic signaling structures at excitatory synapses in brain, where nerve cell communication occurs. Spine size and density reflect the efficiency of signaling through synapses. The average spine size and density in select brain areas are reduced in Alzheimer's disease (AD), correlating with levels of Aβ and reduced synapse efficacy. Here, we tested a core hexapeptide from Aβ, the AβCore, previously shown to be neuroprotective against Aβ-induced compromise of synaptic function, for its ability to rescue Aβ-induced reductions in spine size and density. The results support structural preservation of spines by the AβCore in the presence of Aβ as a correlate of preserved spine function. These findings have implications for application of the AβCore as an AD therapeutic.

## Introduction

Dendritic spines are specialized postsynaptic signaling compartments in excitatory neurons, which undergo dynamic structural changes with alterations in synaptic strength (Matsuzaki et al., 2001, 2004; Yuste and Bonhoeffer, 2001; Bourne and Harris, 2008; von Bohlen Und Halbach, 2009; Yuste, 2011). The dynamic changes in the morphology and composition of dendritic spines, which are central to memory and learning processes (De Roo et al., 2008), are thus regulated by synaptic input from incoming axons. Increased synaptic strength is strongly correlated with larger spines, which have correspondingly larger postsynaptic densities (PSDs; Arellano et al., 2007; Borczyk et al., 2019) and more glutamate receptors (Borczyk et al., 2019). Large spines are correlated with stabilized circuitry underlying long-term memory; smaller spines, which correlate with weaker synaptic strength, appear to be more dynamic (Grutzendler et al., 2002). Notably, the average size and density of spines are much reduced in a number of neuropathological states (reviewed in Penzes et al., 2011; Forrest et al., 2018), including Alzheimer's disease (AD; Dorostkar et al., 2015), and correlates with reduced glutamate receptor densities, synaptic depression, and memory deficits (Hsieh et al., 2006; Knobloch and Mansuy, 2008). In AD, structural and functional alterations in presynaptic terminals also precede the eventual synapse loss.

Spine size dynamics are driven by a balance of regulators that act on the branched actin cytoskeleton in the spine head (Soria Fregozo and Pérez Vega, 2012; Bucher et al., 2020). Key regulators of spine actin dynamics include drebrin, α-actinin, myosins V and VI, Arp2/3, cofilin, Aip1, and Ca^2+^ (reviewed in Pelucchi et al., 2020). Drebrin is an actin-binding protein uniquely concentrated in spines (Aoki et al., 2005; Koganezawa et al., 2017) where it regulates other actin-binding proteins (Hayashi et al., 1996), in particular the myosins (Ishikawa et al., 1994; Shirao et al., 2017), leading to spine stabilization. Drebrin expression in spines is downregulated in AD and AD pathology murine models (Harigaya et al., 1996; Shim and Lubec, 2002; Shirao et al., 2017), correlating with spine shrinkage and loss in the principal areas affected by AD-related pathology, namely, the hippocampus and cortex. These changes correlate with altered synaptic connectivity and plasticity in individuals with AD, further supporting the hypothesis that synaptic dysfunction precedes synaptic and neuronal death (Penzes et al., 2011).

Ca^2+^ plays a central role both for spine actin dynamics and synaptic strength (Oertner and Matus, 2005; Borovac et al., 2018), with CaM kinase II as a primary mediator of the impact of changes in spine Ca^2+^ (Yasuda et al., 2022). In the resting state of the spine, the β subunit of the CaM kinase II interacts with a spine protein complex that includes actin and drebrin, and with synaptic stimulation CaM kinase II is released from the complex to associate with the PSD (Zalcman et al., 2018). In addition, spine size drives postsynaptic Ca^2+^ regulation, with large spines having more stable [Ca^2+^]i, whereas small spines have more dynamic [Ca^2+^]i, correlated with synaptic plasticity (Segal, 2010). Thus, spine size is related both to structural impact on synaptic transmission and regulation of postsynaptic signaling.

Elevated levels of beta amyloid (Aβ) have been correlated with reduced spine density in vivo, as observed for AD as well as model systems for AD pathology (Davies et al., 1987; el Hachimi and Foncin, 1990; Knobloch and Mansuy, 2008; Perez-Cruz et al., 2011). As noted, prolonged exposure of neurons to Aβ in vitro has also been shown to directly alter spine density and spine size (Calabrese et al., 2007). Here, we examined the ability of the neuroprotective AβCore hexapeptide (Forest et al., 2018, 2021) to prevent Aβ-induced reductions in spine size and density in primary mouse hippocampal neuron cultures. The findings have implications for the structural basis for the neuroprotective action of the AβCore on full-length Aβ-induced deficits in synaptic plasticity and fear memory (Forest et al., 2018).

## Materials and Methods

### Mouse primary hippocampal neurons

**Figure 1.**
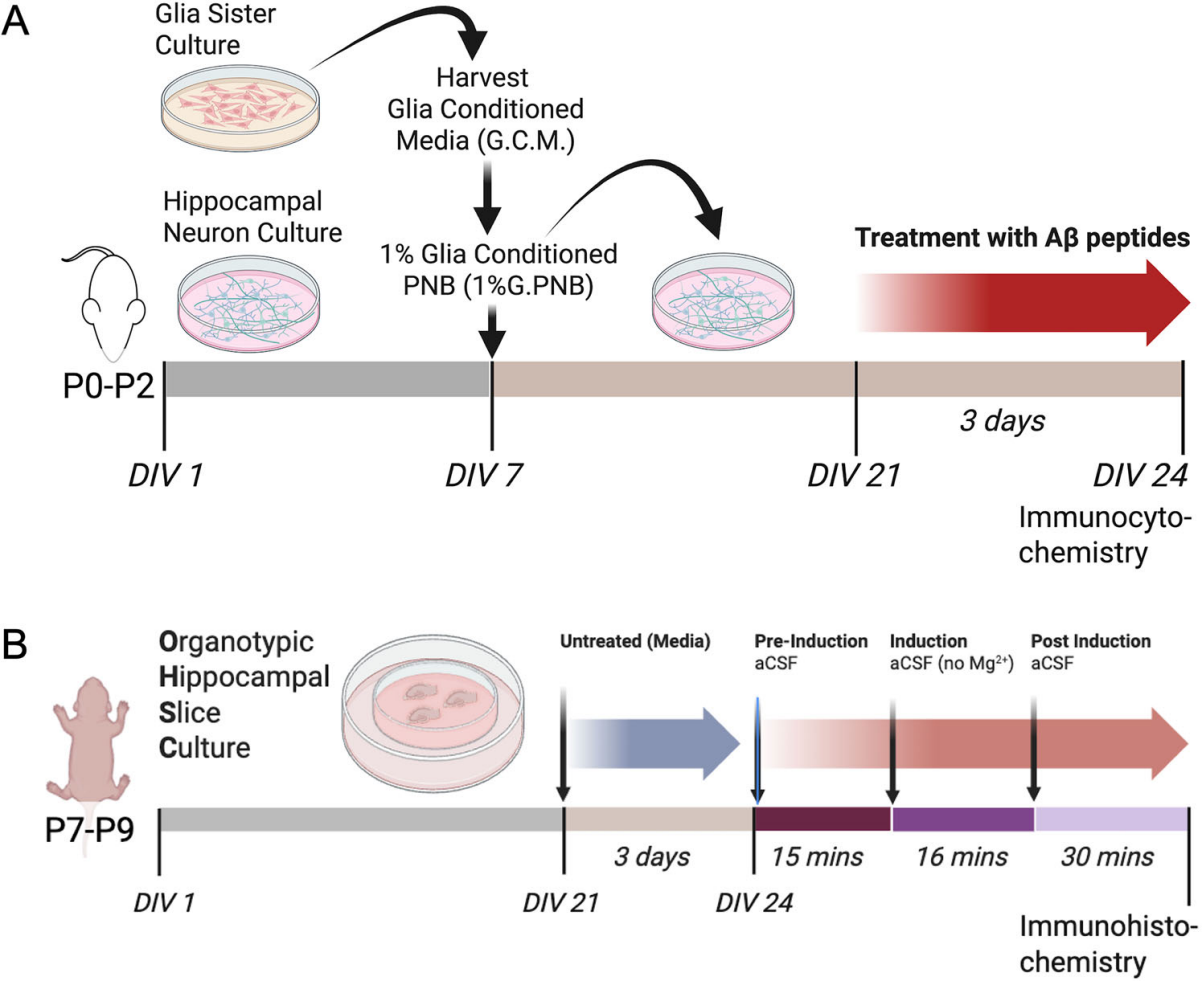
Timelines for hippocampal neuron and organotypic slice culture preparations and treatment. ***A***, hippocampal neuron cultures; ***B***, organotypic hippocampal slice cultures. The 21 DIV is based on previous optimization of neuronal cultures for dendritic spine development (Papa et al., 1995). Viability and reproducibility of neuronal cultures were assessed morphologically (cell density, soma size, and network complexity) by imaging daily during the 21 d preculture period. Created in BioRender. https://BioRender.com/qncvzq1.

Mouse primary hippocampal neuron cultures were prepared from neonatal mouse pups (0–2 d old) of either gender (roughly equivalent numbers) obtained from established colonies of wild-type mice (C57BL/6J, RRID:IMSR_JAX:000664) in the John A. Burns School of Medicine AAALAC-accredited Vivarium, as described (Fig. 1*A*; Seibenhener and Wooten, 2012; Cheng and Yakel, 2015; Forest et al., 2018, 2021). Animal procedures were compliant with NIH and Society for Neuroscience guidelines for using vertebrate animals in neuroscience research under a University of Hawai'i Institutional Animal Care and Use Committee-approved protocol (IACUC Ethical approval reference: 16-2282). Mouse brains were removed into ice-cold Neurobasal A medium (NB) containing B-27 Plus supplement, 5% fetal bovine serum, GlutaMAX-I (1× final concentration), and gentamicin (supplemented NB). Hippocampi were then isolated under a stereomicroscope. The hippocampi were minced and then digested with papain (Worthington, LS003126) in Hanks buffer with 10 mM cysteine at 37°C for 15 min. The preparations were washed by low-speed centrifugation (645 × *g* for 3 min) in supplemented NB. The cells were dissociated from the pellet using sequential trituration with polished Pasteur pipettes of decreasing diameter and collected by low-speed centrifugation (645 × *g* for 3 min). The dissociated cells were preplated in standard tissue culture dishes to remove adherent non-neuronal cells (glia; fibroblasts) for 3–10 min. The neuron-enriched supernatant was plated onto Cell Tak-coated coverslips (Fisher, 354240) in supplemented NB. The cultures were maintained in serum-free NB medium containing B-27 Plus and gentamicin, and for 3D imaging in CultureOne, for 5–6 d, then switched to 10% mixed glia-conditioned media (GCM) in serum-free, supplemented NB until DIV 21 prior to treatment. GCM was prepared from sister primary mixed glia cultures (courtesy of Dr. Megan Lantz) by incubation in serum-free NB media for 1 d. The collected GCM was sterile-filtered and stored as frozen aliquots until use.

### Actin-GFP transduction of hippocampal neuron cultures

Primary hippocampal neuron cultures at 26 DIV were transduced with BacMam Actin-GFP (1:20 dilution in media; Invitrogen C10582, Lot 2303214) for 2 d, followed by treatment or not with full-length Aβ.

### Organotypic hippocampal slice cultures

Organotypic hippocampal slice cultures (OHSCs) were prepared from brains removed from 7–9 d-old C57BL/6J mouse pups (Mewes et al., 2012; Grabiec et al., 2017; Fig. 1*B*). Animal procedures were compliant with NIH and Society for Neuroscience guidelines for using vertebrate animals in neuroscience research under a University of Hawai'i Institutional Animal Care and Use Committee-approved protocol (IACUC Ethical approval reference: 16-2282). Brains were placed into ice-cold dissection media containing 50% modified essential media (MEM), 50% Hanks' buffered salt solution (HBSS), 2 mM glutamine (GlutaMAX-I), 1% antibiotic-antimycotic, 26.6 mM HEPES, 10 mM glucose, and 5 mM MgSO_4_. Transverse brain slices of 275 μm thickness were obtained using a Leica Vibrating Microtome (Leica, VT1200s) and quickly transferred to sterile glass petri dishes. The hippocampi were carefully isolated and plated onto porous membrane inserts (Millipore, PIC0M03050) in 6-well plates with prewarmed OHSC culture media containing 50% MEM, 25% basal medium eagle (BME), 25% heat-inactivated horse serum, 2 mM glutamine (GlutaMAX-I), 1% antibiotic-antimycotic, 0.5 mM ascorbic acid, 0.05% insulin, 26.6 mM HEPES, 0.65% glucose, and 2.5 mM MgSO_4_. OHSC culture media in the well below the insert was changed 24 h after plating and then every other day for 21 d.

### Chemical LTP

**Figure 2.**
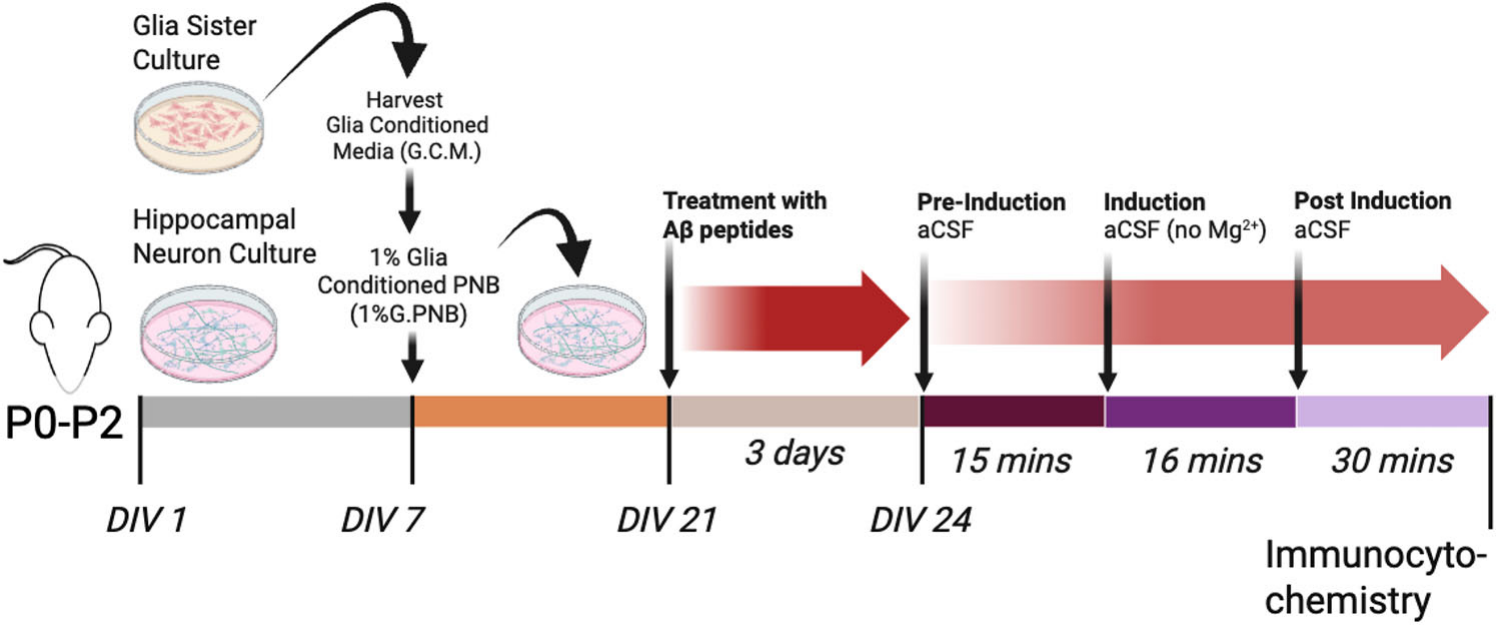
Timeline for induction of cLTP in hippocampal neuron culture preparations. Created in BioRender. https://BioRender.com/augfmxp.

For stimulation via chemical LTP (cLTP), solutions were prepared in artificial cerebrospinal fluid (ACSF; in mM: 130 NaCl, 3.5 KCl, 10 glucose, 1.25 NaH_2_PO_4_, 2.0 CaCl_2_, 1.5 MgSO_4_, and 24 NaHCO3) or modified ACSF (ACSF without MgSO_4_) bubbled with 95% O_2_/5% CO_2_ for 15 min. The culture media was aspirated, and cultures were pretreated in warmed ACSF with or without (control) 6 μM 2-chloroadenosine (2-CADO) for 15 min at 37°C/95% O_2_/5% CO_2_. Chemical LTP was then induced (Figs. 1*A*, 2) by application of 50 μM forskolin, 0.1 μM rolipram, and 50 μM picrotoxin or DMSO (control) in modified ACSF (Mg^2+^-free without 2-CADO) for 16 min at 37°C in 95% O_2_/5% CO_2_ (Otmakhov et al., 2004a,b; Makino and Malinow, 2009). After the 16 min of stimulation, cultures were placed in ACSF and fixed at 30 min post stimulation. Note that forskolin and rolipram strongly activates the synaptic cAMP-dependent pathways, while picrotoxin induces high-frequency firing, inducing LTP similar to that observed using electrophysiological stimulation. Inducing LTP via bath application by chemical reagents (cLTP) affects all cells in the culture.

### Aβ preparation

Full-length Aβ_1–42_ (Aβ42) was obtained as hydrochloride salts from American Peptide/BACHEM (catalog #4045866-1000). The AβCore (Aβ_1–15_), YEVHHQ, and the inactive substituted AβCore, SEVAAQ, were custom-ordered from Peptide 2.0. [There is no evidence that the AβCore influences Aβ oligomer formation (Forest et al., 2018).] All peptides were synthesized and isolated at >98% purity, as assessed by mass spectrometry. Aβ42 and the AβCore were dissolved in double-distilled water and used at various final concentrations as per previous studies (Forest et al., 2018, 2021). Full solubilization of Aβ42 required brief bath sonication.

### Immunostaining

Primary hippocampal neurons were fixed with 4% paraformaldehyde for 40 min and then rinsed twice with 1× PBS for 15 min. Hippocampal neurons were then permeabilized with 0.1% Triton X-100 in 1× PBS and incubated for 30 min and then rinsed twice with 1× PBS for 15 min. Samples were then blocked with 5% goat serum/0.1% TWEEN in 1× PBS for 30 min and then washed once with 1× PBS for 15 min with gentle rocking. Cells were then incubated with mouse anti-Drebrin antibody [Abcam [M2F6] (ab12350), dilution 1:1,000] or anti-postsynaptic density 95 (PSD95; Thermo Fisher Scientific, #MA1-045; 1:400), anti-MAP2 antibody (Millipore, #MAB3418; 1:250 or Thermo Fisher Scientific, #PA1-10005; 1:1,000) and, where studied, anti-synaptophysin (Thermo Fisher Scientific, #PA1-1043; 1:500) in 5% goat serum/0.1% TWEEN in 1× PBS for 18 h at 4°C. Cells were then washed three times with 1× PBS for 15 min per wash. Cells were then incubated with matched fluorophore-conjugated secondaries (1:1,000): Goat anti-Chicken IgG Alexa Fluor 405 (Thermo Fisher Scientific, #A48260); Goat anti-Mouse IgG Alexa Fluor 488 (Molecular Probes, #A-11029); Goat anti-Guinea Pig IgG Alexa Fluor 555 (Thermo Fisher Scientific, #A-21435); Goat anti-Rabbit IgG Alexa Fluor 633 (Thermo Fisher Scientific, #A-21071); Alexa Fluor 633 goat anti-mouse IgG (Thermo Fisher Scientific, #A21052; Lot: 1622583) in 5% goat serum/0.1% TWEEN in 1× PBS for 1 h. As controls, separate replicates were incubated with secondary antibodies only. Cells were then washed with 5% goat serum/0.1% TWEEN in 1× PBS for 15 min with gentle rocking. Cells were then washed twice with 1× PBS for 15 min. Up to and including mounting, all steps were performed in the dark. Labeled cells were then mounted onto VWR VistaVision Microscope Slides (#16004-368; Lot: 4589202) with VectaShield Anti-fade Mounting Medium with DAPI (#H-1200; Lot: ZB1130) and imaged using a Leica TCS SP8 confocal microscope with HyVolution using oil immersion 40× and 63× objectives.

### Spine morphology and number

#### Cross-sectional (2D) analysis

Following immunostaining of mouse primary hippocampal neurons with the dendritic marker MAP2 and the actin-binding protein Drebrin or actin labeling with BacMam actin-GFP, images were taken using the Leica SP8 HyVolution confocal microscope at a final magnification of 400× in comparison with replicates incubated with secondary antibodies only to set the signal-to-background threshold. Images were analyzed using ImageJ with the BioFormats plugin. The following criteria were applied to determine which spines were counted and measured. Puncta were categorized as spines if they were in close proximity to a dendrite of uniform MAP2 staining, whether a spine neck was evident or not, within a distance not greater than the width of the dendrite. Second, for 2D imaging only spines perpendicular to the dendrite were counted to minimize spine area determination error, as determined by position in *Z*-stacked images. Thus, spines appearing to be offset from perpendicular, as evident in *Z*-stacks, were not counted. Sections with overlapping processes were excluded from counts and measurements. Spines that met these criteria were outlined using the “freehand selection” function in FIJI (ImageJ) and were measured for cross-sectional spine area and number per unit length of dendrite using area integration and count features. Data for each were exported into Excel, ranked and plotted (scatter over violin or box and whiskers) and then converted to fractional distribution plots to allow normalized comparisons of spine populations from different treatment conditions. Spine area was used as proxy for spine volume, to indicate spine size. Spines were not categorized into morphologies. Note that while expression of Drebrin has been found to be altered by Aβ_1−42_ treatment (Extended Data Fig. 5-2), consistent with previous reports (Lacor et al., 2007; Ishizuka and Hanamura, 2017), differences in signal intensity did not impact determination of spine size or number.

#### Volumetric (3D) analysis

**Figure 3.**
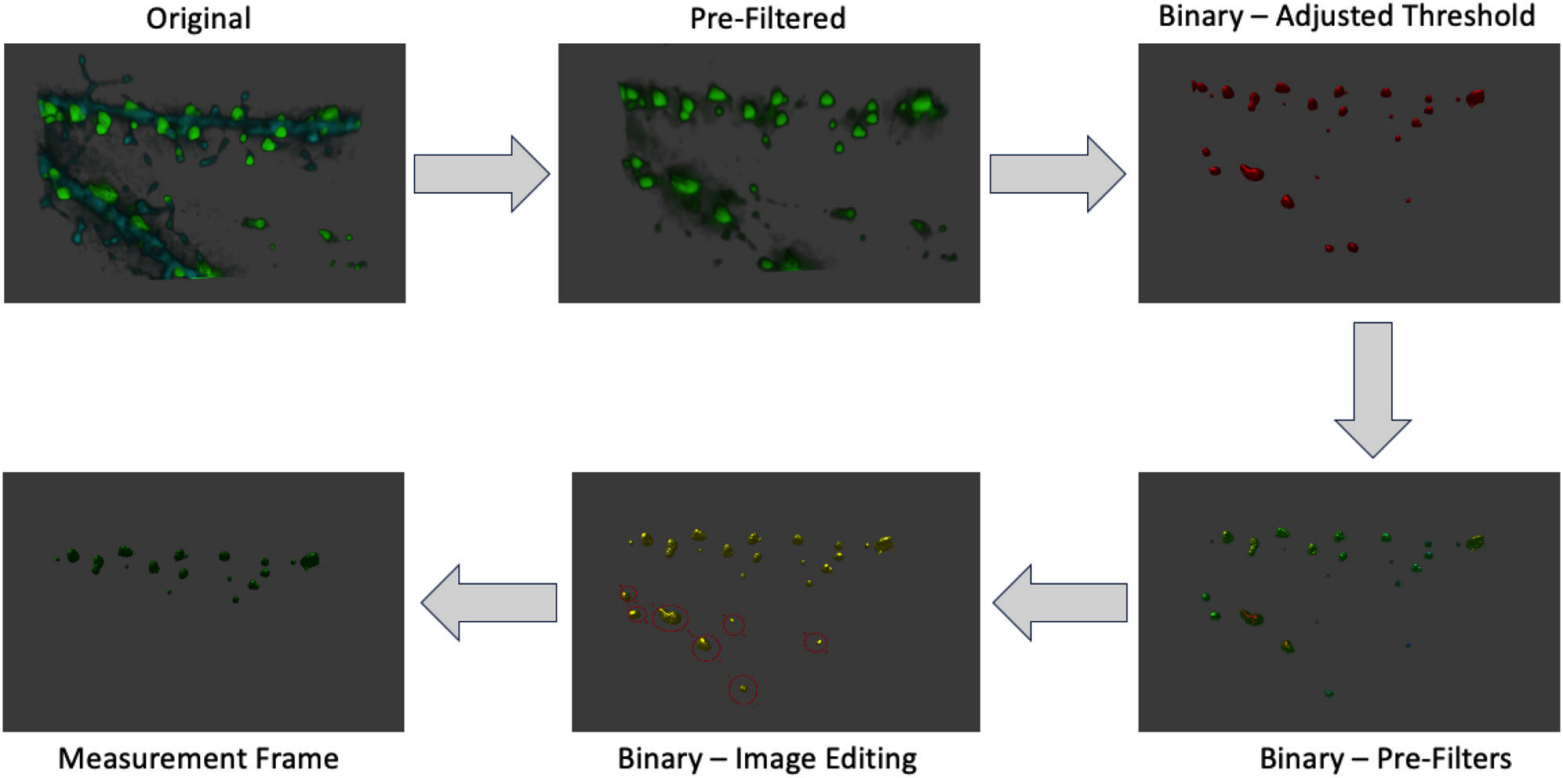
Overview of LASX 3D Analysis workflow.

3D rendering and analysis for both single-channel and multichannel imaging of immunostained hippocampal neuron dendrites were performed using the Leica DMi8 Thunder widefield microscopy system. The Leica Application Suite X (LAS X) 3D analysis software module allowed efficient interrogation of thousands of imaged spines (Fig. 3) using an automated, unbiased approach. In addition, the 3D reconstruction within the Leica LAS X program does not require manual segmentation via each *Z*-stack, facilitating unbiased batch analysis. A preview of the entire coverslip was done on the UV405 channel (MAP2 staining) using the 20× high NA objective (NA, 0.8). Region of interest (ROI) images were then taken with a 100× oil objective at 2,048 × 2,048 size, 16 bit, with entire cells being a 4–9 tile image. All images were taken with optimal system *Z*-step of 0.18 μm, for a stack of 10.15 μm thick to ensure all dendrites were within the image. Post-processing deconvolution was done using Leica LAS X Small Volume Thunder/Lightening deconvolution under optimal settings for all images. Three to five neurons were imaged from different quadrants of the coverslip per treatment, and five dendrites per neuron per treatment were analyzed from three to five sets of experiments, unless otherwise stated. Single-channel and multichannel analysis were performed using the same filters, thresholds, and preprocessing methods.

#### Synapse number

**Figure 4.**
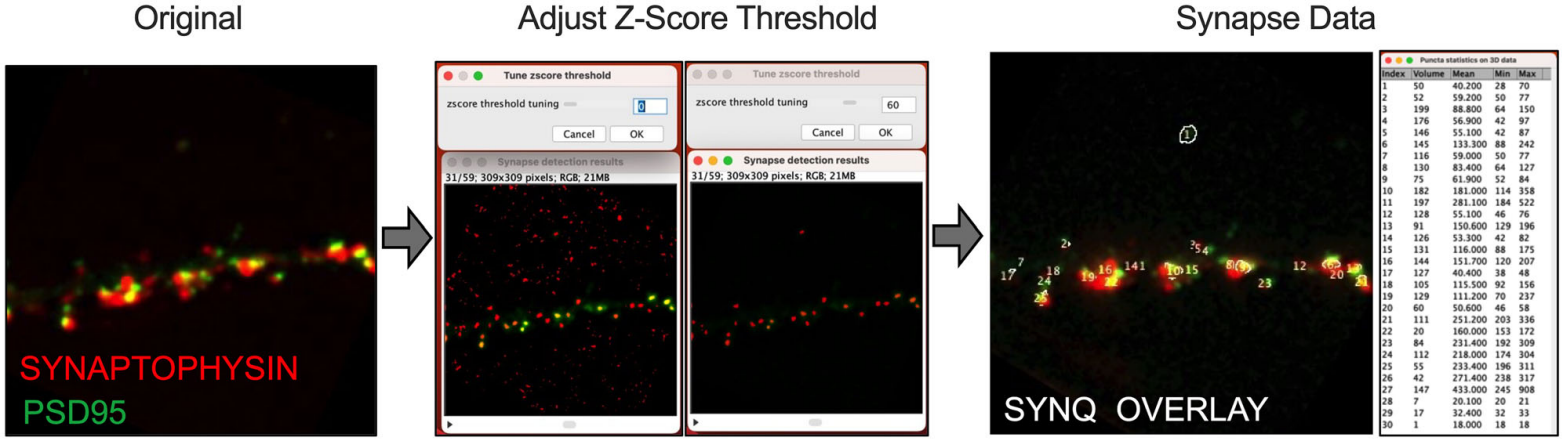
SynQuant workflow for synapse counts. SynQuant analysis of 3D images accessed as a FIJI plugin. Left panel, Original input of two channels for pre- (synaptophysin) and postsynaptic (PSD95) immunostaining of an example hippocampal neuron dendrite. Middle panel, Adjustment of *Z*-score threshold. Right panel, Example final synapse detection and quantified parameters.

Synapse number was assessed using the automatic probability-principled synapse detection plugin SynQuant in FIJI (Wang et al., 2020). The pre- and postsynaptic markers used were the proteins synaptophysin and PSD95, respectively, visualized with the Leica Thunder via immunocytochemistry. SynQuant uses neighborhood pixel correction and order statistics to provide an unbiased score to each potential synapse region. SynQuant was able to detect and quantify synapses from heterogeneous images (Fig. 4).

### Statistical analysis

Experiments were repeated a minimum of three times. No power analysis was performed, as the sample size and replicates were based on previously published studies. Mean spine areas in 2D imaging are displayed as mean ± SEM (standard error of mean) because median values derived from normalized population distributions were averaged. For concentration-dependent impact of the AβCore, data are presented as median ± SEM from distribution plots. Slope values were generated using 40–60% fractional distributions for the spine area and 25–75% fractional distributions for spines per micrometer using regression analysis performed in Excel. Dunn's pairwise post hoc Kruskal–Wallis test was applied to analyze the difference in medians in the integrated fluorescence (F*) area values across the population of spines (violin or box and whisker plots) or in the normalized rank fractional distribution data for spine area and density, using α = 0.05 as the threshold for significance. Mean PSD volumes (3D imaging) are displayed as mean ± 95% confidence intervals (CIs). After testing for normality (Gaussian) of distribution using Kolmogorov–Smirnov tests, two-tailed Student’s *t* tests of pairwise comparisons were conducted using 5–95% CIs, while multiple conditions were assessed using one-way ANOVA followed by Tukey’s post hoc pairwise comparisons. A *p* value of <0.05 was considered the minimum for significance to reject the null hypothesis. Data figures were generated using Prism (GraphPad v10.5.0; RRID:SCR_002798).

## Results

### Impact of AβCore on Aβ-induced reduction in spine cross-sectional size and number

**Figure 5.**
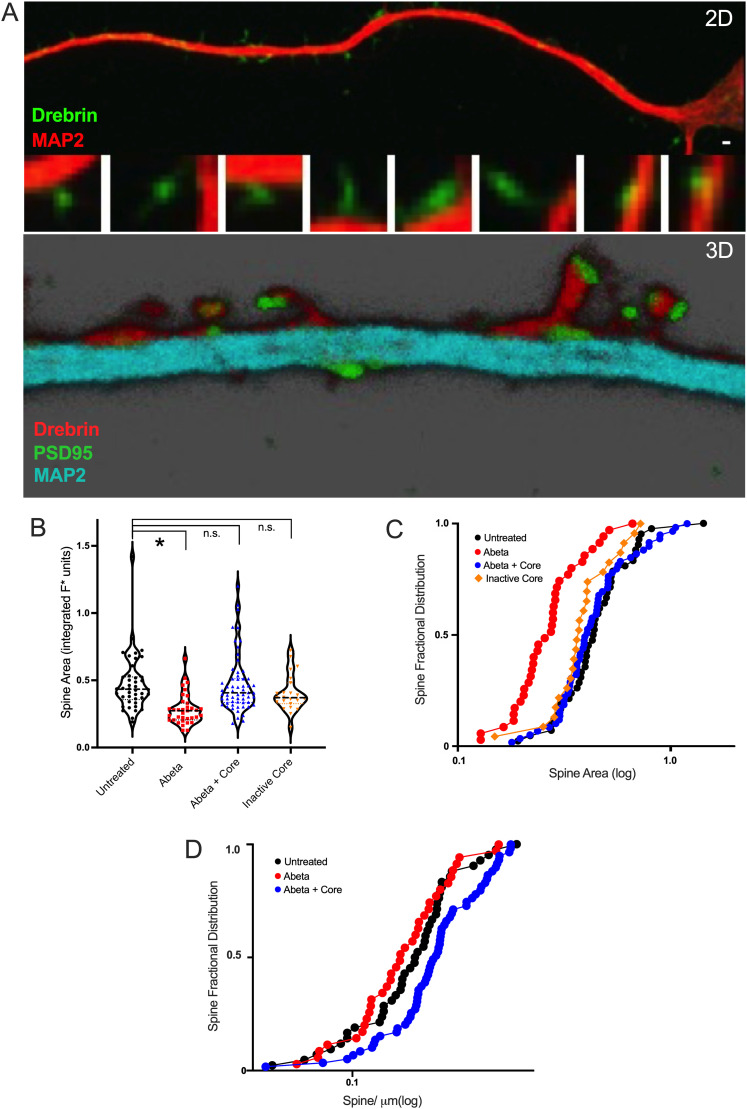
The AβCore prevents the Aβ42-induced reduction in dendritic spine size and density. ***A***, 2D confocal image (top panel) and 3D imaging (bottom panel: 3D projection) of immunostaining of mouse hippocampal neuron cultures for Drebrin (2D: green; 3D: red), MAP2 (2D: red; 3D: cyan) and PSD95 (3D: green) as described under Materials and Methods. Cultures were established as described under Materials and Methods. Insets in the top panel are magnified views of spines present along the dendrite shown in the top image. Top, scale bar, 1 µm. ***B***, Treatment with 0.5 µM Aβ_1–42_ (Abeta) reduced spine cross-sectional area as visualized in Drebrin-immunostained spines and verified independently by visualizing spines with actin-GFP as presented in Extended Data Figure 5-1. Cotreatment with the AβCore (YEVHHQ; Core) at 0.1 µM normalized the spine area altered by Aβ_1−42_ (Abeta) treatment for 3 d. The spine area distribution for treatment with the Inactive Core (SEVAAQ; Forest et al., 2018) was provided as a separate control from the untreated condition. Each point represents the averaged spine size for a given dendrite. Medians in violin plots denoted by hashed lines. Post hoc pairwise statistical comparison versus untreated condition using Dunn's test following Kruskal–Wallis analysis: *p < 0.001; n.s. not significant. ***C***, Replotting the spine cross-sectional area data shown in *B* as rank-ordered fractional distributions indicates that the impact of Aβ_1–42_ (Abeta) to reduce the spine area was relatively uniform across the spine population, as was the impact of cotreatment with the AβCore (Core). ***D***, Treatment with Aβ_1–42_ (Abeta) reduced the density across the rank-ordered fractional distribution of dendritic spines, which correlates with reduced levels of spine Drebrin (Lacor et al., 2007; Ishizuka and Hanamura, 2017), as verified in Extended Data Figure 5-2. The presence of the AβCore (Core) normalized the fractional distribution of spine density altered by Aβ42. Each point represents averaged spine density per dendrite. Sample sizes: 551 spines from untreated cultures; 324 spines from cultures treated with Aβ42; 405 spines from Aβ42 + AβCore.

Drebrin was used as a marker for changes in dendritic spines in murine models exhibiting AD-like pathology (Harigaya et al., 1996; Shim and Lubec, 2002; Shirao et al., 2017), as its presence across the spine is relatively uniform. To localize the structures to dendrites, coimmunostaining for the dendrite microtubule protein, MAP2, to visualize the entire dendrite was performed, revealing clear immunolabeling of drebrin in spines on identified dendrites in primary mouse hippocampal neurons (Fig. 5*A*). The range of structures along the dendrites immunostained with anti-drebrin are consistent with various types of spines structures, from stubby to thin to mushroom-like (Fig. 5*A*, top magnified insets), across a size range typical for spines (Harris and Stevens, 1989; Calabrese et al., 2006). The various spine morphologies, as categorized, are however not discreetly stable, but rather component to a dynamic continuum (Arellano et al., 2007). Consequently, the full range of spine sizes across the spine population was subsequently assessed via fractional distribution analysis.

10.1523/ENEURO.0044-25.2025.f5-1 Figure 5-1 **Aβ42-induced alterations in dendrite spine parameters of hippocampal neurons visualized with Actin-GFP. *A***, Representative dendrites of neurons labeled with Actin-GFP using BacMam transduction, following treatment or not with Aβ42 for 1 or 5 days. Graphs are plots of summarized analyses of the number of spines per unit length of dendrite (***B***), spine length: μm (***C***) and spine area: μm^2^ (***D***). Download Figure 5-1, TIF file.

10.1523/ENEURO.0044-25.2025.f5-2 Figure 5-2 **Impact of AβCore on Aβ42-induced changes in Drebrin expression**. Rescue of Aβ-induced reduction of drebrin immunocytochemical expression (mean integrated fluorescent intensity values in arbitrary units) in identified dendritic spines by co-treatment with the AβCore (Core). Data are means +/- SD; Sample sizes (# of spines analyzed): 551 Untreated; 543 Inactive Core; 324 Aβ; 405 Aβ + Core. *p=0.03 Aβ vs. Untreated by post hoc comparison following ANOVA. Download Figure 5-2, TIF file.

Consistent with previous results using a range of probes (Lacor, et al., 2007), treatment with pathological levels Aβ_1–42_ (Aβ42, high nM–µM range) resulted in a reduction in dendritic spine size and spine number when compared with the spines in untreated cultures (Fig. 5). The reduced spine area is an indication of reduced spine volume, evidence for shrinkage of dendritic spines. Aβ42 treatment resulted in a 61.5% reduction in median spine size compared with the untreated condition (Fig. 5*B*; Dunn's test: *p < 0001). Spine population analysis using normalized rank-ordered distributions revealed that the median value for untreated spines was 0.43 μm, while the 500 nM Aβ42 treatment had a median value of 0.27 μm (Fig. 5*C*; Dunn's test: *p* < 0.0001). Upon cotreatment with the AβCore, the dendritic spine shrinkage induced by Aβ42 was reversed to a median value of 0.41 μm, not significantly different from average spine size in untreated cultures or cultures treated with the inactive AβCore (Fig. 5*B*; Dunn's test: *p* > 0.99). That the slopes of the spine distributions across the different treatments were similar (Fig. 5*C*), with the distributions shifting depending on the treatment, indicates the impact of the various conditions on spine size was relatively uniform across the spine population of varying types from thin to mushroom-like. As an independent assessment of Aβ42 regulation of spine size and density, actin-GFP was expressed in the hippocampal neurons via actin-GFP-BacMam vector transduction to label the dendritic spine cytoskeleton and Aβ42-induced reduction in dendritic spine size and spine number at 1 and 5 d was confirmed (Extended Data Fig. 5-1).

To investigate the effects of AβCore on the Aβ42-induced dendritic spine density, spines were counted along a measured length of dendrite and then normalized by calculating the number of spines per micrometer. The median value for the untreated cultures was 0.21 spines per micrometer. Aβ42 treatment caused a 16% reduction in the median number of spines per micrometer to a median value of 0.18 spines per micrometer (Fig. 5*D*). There is evidence that Aβ42 treatment-induced dendritic spine loss is regulated through changes in actin stability and dynamics mechanisms (Pelucchi et al., 2020), consistent with reduced spine drebrin (Extended Data Fig. 5-2). Cotreatment with the AβCore resulted in 151% increase in spines per micrometer compared with treatment with Aβ42 only (Dunn's test: *p* < 0.002). The median value for the Aβ42 + AβCore was 0.27 spines per micrometer, somewhat increased compared with that found for spines in untreated cultures. While the impact of the various conditions on spine density was less pronounced than that seen for spine size, the similar slopes observed for these conditions would, once again, indicate uniform impacts across the spine populations.

### Time course for Aβ-induced changes in dendritic spine area and density

**Figure 6.**
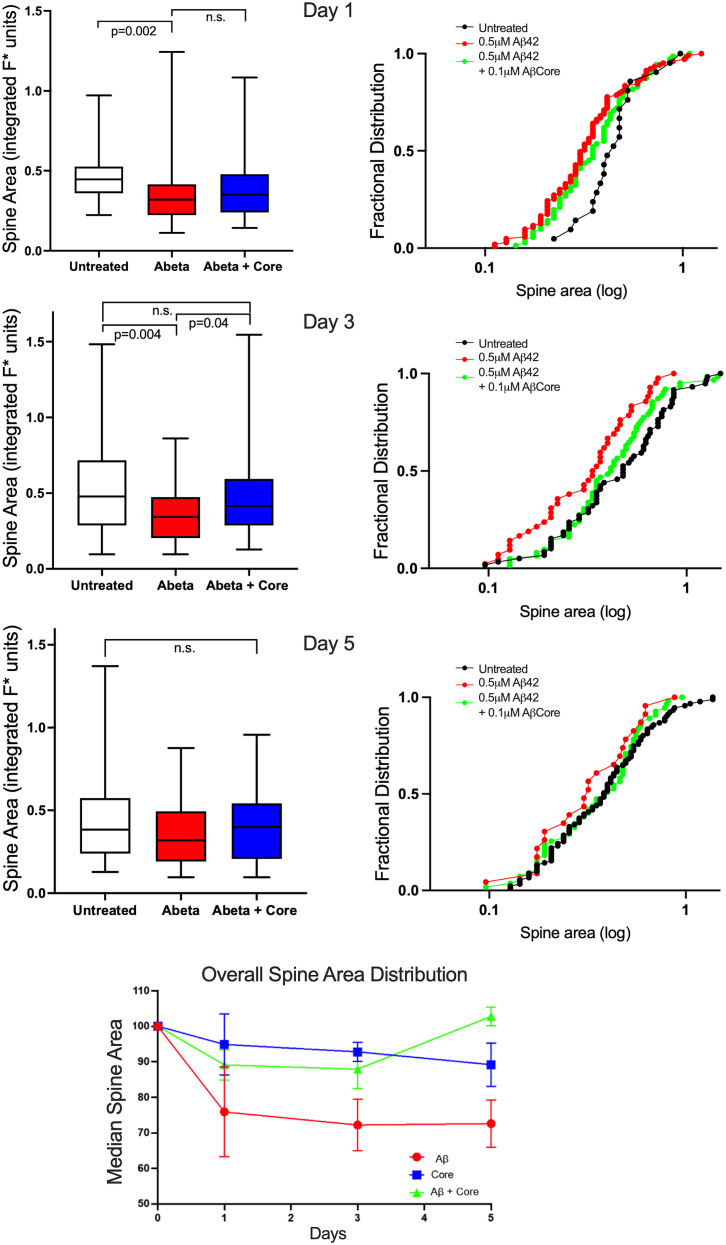
Time course of the impact of Aβ42 without or with the AβCore on dendritic spine area of primary hippocampal neurons. Left graphs, Time course of the impact of the AβCore (Core) at 0.1 µM on the spine area altered by 0.5 µM Aβ_1–42_ (Abeta) treatment for 1–3 d. Medians in box-and-whisker plots denoted by lines. Post hoc pairwise statistical comparison versus untreated condition using Dunn's test following Kruskal–Wallis analysis: *p* values as listed; n.s. not significant. Right graphs, Replotting spine area as rank-ordered fractional distributions for 1, 3, and 5 d of treatment as noted in *A*, to allow comparison of spine populations. Bottom plot, Overall spine area distribution medians for 1, 3, and 5 d. Points are mean values of rank-order median ± SD (*n* = 3 experiments). Overall sample size: a total of 2,211 dendritic spines measured. For the number of spines assessed for each condition on each day, see Table 1.

Spine area and density in primary hippocampal neurons were analyzed over 1, 3, and 5 d under various treatment conditions (Fig. 6; Tables 1, 2). Treatment with Aβ42 led to a 26% reduction in mean spine area relative to controls by Day 1, plateauing thereafter through Days 3 and 5 (Fig. 6, Table 1), indicating a relatively rapid impact of Aβ42 on spine size. In contrast, the impact of Aβ42 on spine density was more delayed, with a significant reduction only evident at Day 3 (Table 2). Cotreatment of Aβ42 with the AβCore rescued the spine size to ∼88% of the control mean spine area at Days 1 and 3, with full return to control spine area values by Day 5 (Fig. 6, Table 1; Dunn's test: *p* > 0.99). Similarly, the AβCore normalized the Aβ42 reduction in spine density at Day 5. The AβCore alone had little to no impact on spine area (<10% change relative to controls) across the time frame studied (Fig. 6) but appeared to increase spine density at earlier time points (Table 2).

**Table 1.** Summarized impact of Aβ peptides on mean spine area of primary hippocampal neurons over 1, 3, and 5 d (3 experiments)

	Treatment	# of dendritic spines	Mean spine area (µm)	% untreated	*p* value
Day 1	Untreated	139	0.420 ± 0.035	**–**	
	0.5 µM Aβ42	207	0.308 ± 0.037	73.5	<0.001
	0.1 µM AβCore	225	0.391 ± 0.008	93.2	<0.001
	Aβ42 + AβCore	227	0.372 ± 0.029	88.6	<0.001
Day 3	Untreated	159	0.473 ± 0.005	–	
	0.5 µM Aβ42	111	0.340 ± 0.039	72.0	<0.001
	0.1 µM AβCore	213	0.438 ± 0.012	92.7	n.s.
	Aβ42 + AβCore	147	0.415 ± 0.027	87.7	<0.001
Day 5	Untreated	224	0.430 ± 0.027	–	
	0.5 µM Aβ42	105	0.308 ± 0.019	71.7	0.036
	0.1 µM AβCore	259	0.388 ± 0.055	90.2	<0.001
	Aβ42 + AβCore	195	0.441 ± 0.021	102.5	n.s

Combined peptide concentrations: 0.5 µM Aβ42 + 0.1 µM AβCore. # of dendritic spines are the number of spines measured. # of dendrites measured for each condition ranged from 26–49. Length of dendrite for each condition ranged from 6–15 µm. *p* values are for comparisons to untreated condition.

**Table 2.** Summarized impact of Aβ peptides on mean dendritic spine density (per µm) of primary hippocampal neurons over 1, 3, and 5 d (3 experiments)

	Treatment	# of spines	Mean spine # per µm dendrite	% of untreated	*p* value
Day 1	Untreated	139	0.108 ± 0.062	**–**	
	0.5 µM Aβ42	207	0.104 ± 0.030	96.3	n.s.
	0.1 µM AβCore	225	0.124 ± 0.034	114.8	0.003
	Aβ42 + AβCore	227	0.125 ± 0.047	115.4	0.005
Day 3	Untreated	159	0.106 ± 0.003	–	
	0.5 µM Aβ42	111	0.097 ± 0.038	91.5	0.005
	0.1 µM AβCore	213	0.125 ± 0.043	128.9	0.005
	Aβ42 + AβCore	147	0.117 ± 0.026	120.6	0.035
Day 5	Untreated	224	0.114 ± 0.042	–	
	0.5 µM Aβ42	105	0.078 ± 0.017	68.4	0.002
	0.1 µM AβCore	259	0.123 ± 0.019	107.9	0.004
	Aβ42 + AβCore	195	0.103 ± 0.039	90.4	0.05

# of dendrites for each condition ranged from 26–49. Length of dendrite for each condition ranged from 6 to 15 µm.

To compare the impact of Aβ42 versus AβCore across the population of spines analyzed in each culture over time, the slope values were analyzed as an indicator of shifts in putative spine types (e.g., smaller “thin” vs larger “mushroom-like”). While there appeared to be an increase in the proportion of smaller spines on treatment with Aβ42 for 1 d (Fig. 6, right graphs), yielding a 1.7-fold increase in spine population slope, this was less apparent at Days 3 and 5, reflecting a relatively uniform impact on the spine population across all sizes from relatively small to large. A uniform impact on the spine population also appeared to be the case for cotreatment with AβCore.

### Dose dependency of protection by the AβCore against Aβ-induced reductions in spine size and density

**Figure 7.**
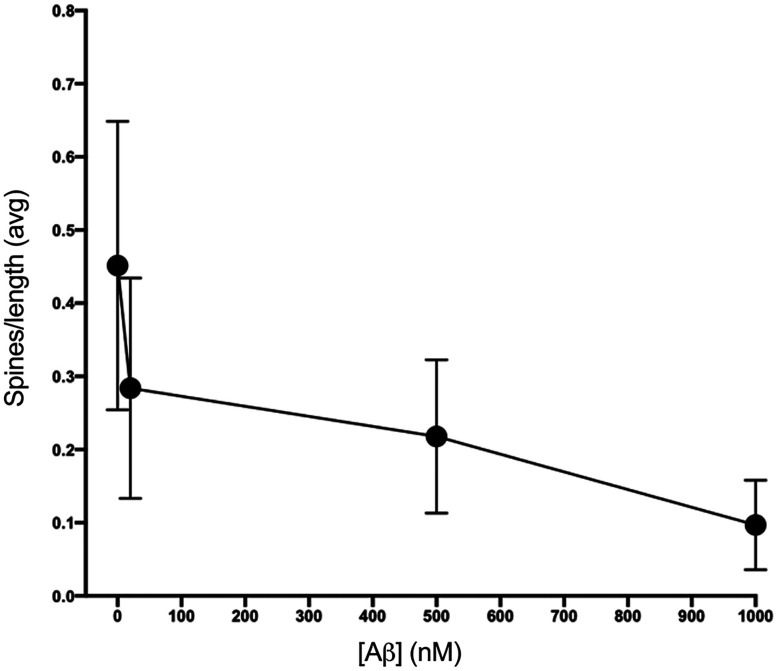
Mean dose–response of the number of dendritic spines per micrometer dendrite length affected by Aβ42 treatment. Sample sizes: No treatment (0)—92 total spines; 20 nM Aβ_1−42_ treatment—216 total spines; 500 nM Aβ_1−42_ treatment—78 total spines; 1,000 nM Aβ_1−42_ treatment—38 total spines. Data are mean ± SD.

**Figure 8.**
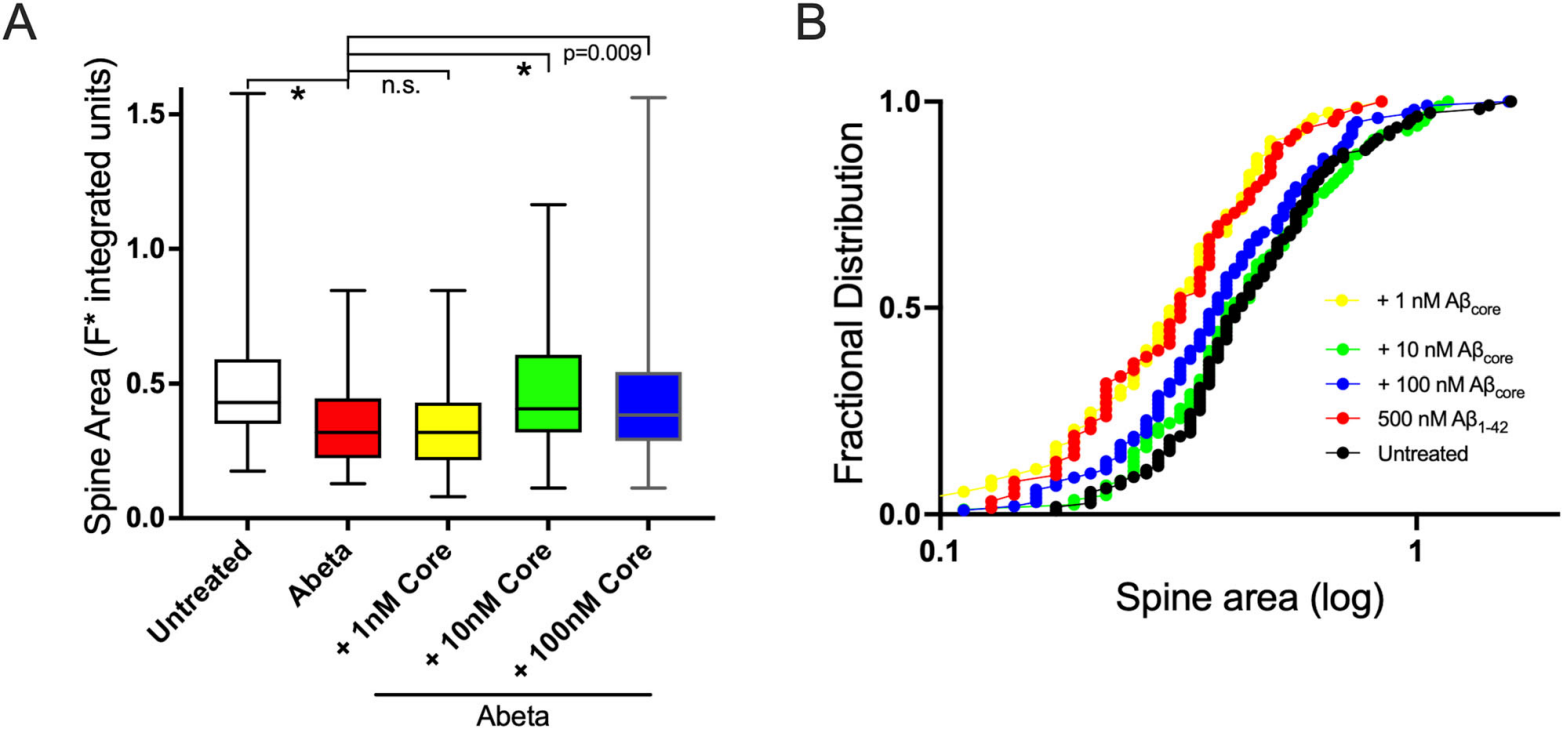
Impact of 1–100 nM AβCore on Aβ42-induced changes in dendritic spine area of primary hippocampal neurons. ***A***, Dose–response of the impact of cotreatment with the AβCore (Core) on the spine area altered by 0.5 µM Aβ_1–42_ (Abeta) treatment. Medians in box-and-whisker plots denoted by lines. Post hoc pairwise statistical comparison versus untreated condition using Dunn's test following Kruskal–Wallis analysis: *p < 0.0001; other *p* values as listed; n.s. not significant. ***B***, Rank-ordered fractional distributions of spines analyzed under the conditions listed in ***A***. The number of spines assessed is listed in Tables 3 and 4.

As expected, Aβ-induced reduction in the spine density was dose dependent, with significant impact down in the nM range (Fig. 7), similar to that seen for oxidative stress-based neurotoxicity (Forest et al., 2018). For comparison, dose–response effects of AβCore prevention of the Aβ42-induced reductions in the spine area and density were investigated. Full rescue of the Aβ-induced reduction of spine size across the spine population was observed down to 10 nM AβCore (Fig. 8, Table 3), while rescue of the reduction in spine density was evident at 1 nM AβCore, particularly for the higher density range of the spine population (Table 4). These results indicate a highly potent action of the protective AβCore peptide against neurotoxic levels of Aβ, particularly in preventing spine loss.

**Table 3.** Summarized data on the concentration dependence of the impact of the Aβcore on spine area (from 3 experiments)

Treatment	# of dendritic spines	Median spine area (µm)	% untreated	*p* value
Untreated	111	0.430 ± 0.024	**–**	
0.5 µM Aβ42	63	0.319 ± 0.02	74.2	<0.001
0.5 µM Aβ42 + 0.1 µM AβCore	101	0.383 ± 0.022	89.1	<0.001
0.5 µM Aβ42 + 10 nM AβCore	86	0.399 ± 0.025	92.8	<0.001
0.5 µM Aβ42 + 1 nM AβCore	73	0.319 ± 0.018	74.2	<0.001

Combined peptide concentrations: 0.5 mM Aβ42 + various concentrations of AβCore. # of dendritic spines are the number of spines measured. Number of dendrites measured ranged from 8 to 13.

**Table 4.** Summarized data for dose-dependent response of Aβcore on # of dendritic spines per µm from hippocampal primary neurons (3 experiments)

Treatment	# of dendritic spines	Median spines per µm	% untreated	*p* value
Untreated	111	0.169 ± 0.024	**–**	
0.5 µM Aβ42	63	0.067 ± 0.005	39.6	0.022
0.5 µM Aβ42 + 0.1 µM AβCore	101	0.148 ± 0.025	87.6	0.007
0.5 µM Aβ42 + 10 nM AβCore	86	0.157 ± 0.016	92.9	0.012
0.5 µM Aβ42 + 1 nM AβCore	73	0.140 ± 0.016	82.8	0.144

Combined peptide concentrations: 0.5 µM Aβ42 + various concentrations of AβCore. # of dendritic spines are the number of spines measured. Number of dendrites measured ranged from 8 to 13.

### Comparison of impact of AβCore on Aβ-induced reduction in postsynaptic density volume

**Figure 9.**
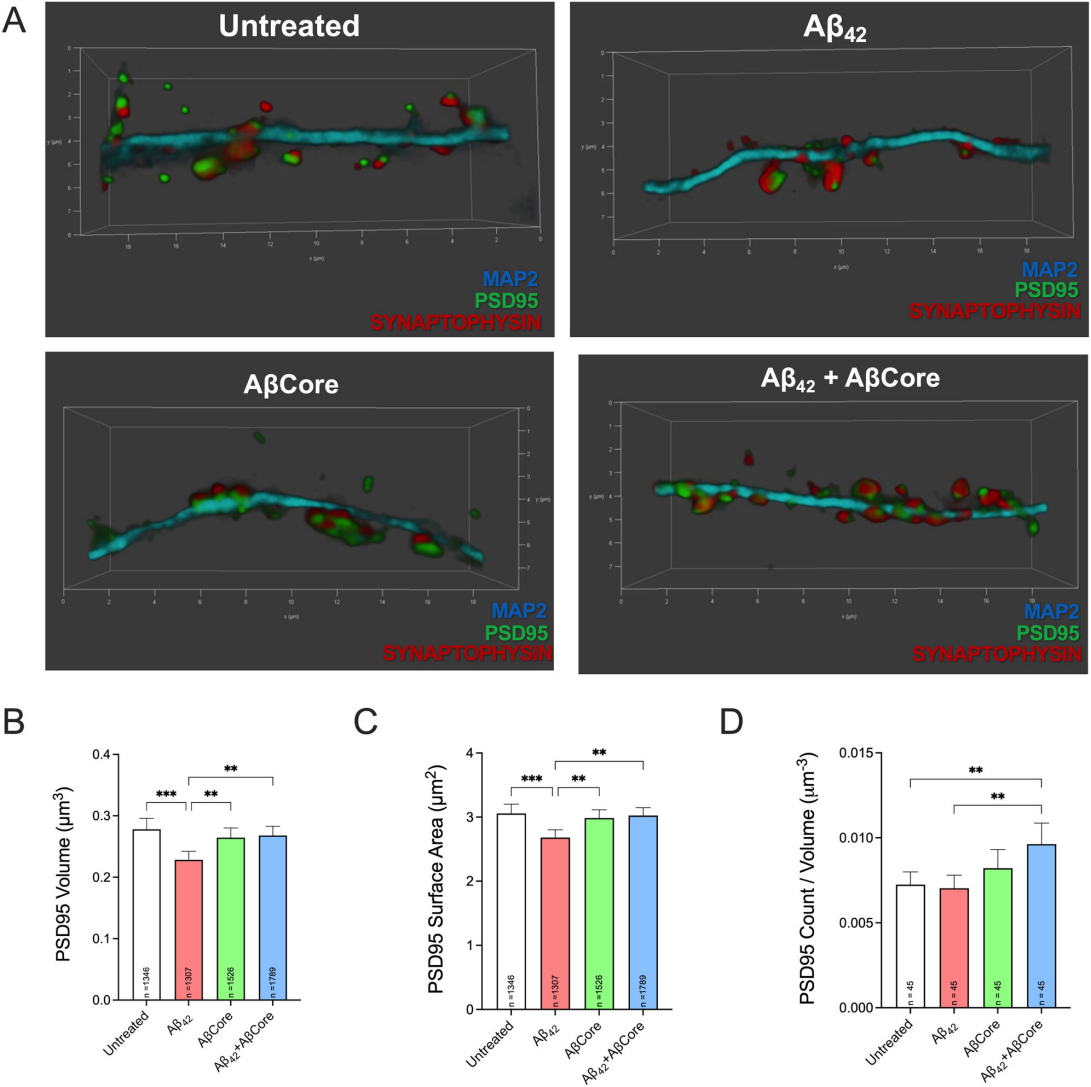
Treatment of hippocampal neurons with the AβCore prevents Aβ42 reduction in postsynaptic density (PSD) volume and surface area. Primary mouse hippocampal cultures (DIV 21 d) were treated or not (untreated) with 1 μM Aβ_42_ or AβCore or both for 3 d. ***A***, Representative 3D images of a dendrite containing visualized for PSD95 on associated spines. Images taken at 100× of secondary and tertiary dendrites, averaging 20 μm in length, immunostained for MAP2 (cyan) for the dendritic shaft, PSD95 (green) for the postsynaptic membrane, and synaptophysin (red) for the presynaptic nerve terminal. All images were deconvoluted and the channels were set to the same intensity range via Leica LASX software for analysis of volume (***B***), surface area (***C***), and count (***D***) of PSDs associated with the identified dendrites. Data are expressed as mean ± 95% confidence interval (CI) with sample size (*n*). Data were analyzed by one-way ANOVA followed by Tukey’s post hoc comparisons: * < 0.05; ** < 0.01; *** < 0.001.

Postsynaptic densities (PSDs) are tightly regulated and contain nanodomains harboring ion channels, neurotransmitter receptors, regulatory proteins, protein kinases, and cell adhesion molecules (Harris and Stevens, 1989). The postsynaptic density 95 (PSD95) protein is one of the major constituents of dendritic spines and has been shown to interact with NMDARs and AMPARs, regulating insertion and diffusion at synaptic sites (Chen et al., 2015; Jeyifous et al., 2016). As a complement to our results of the ability of the AβCore to rescue the Aβ42-induced changes measured using 2D cross-sectional analysis (Figs. 5–8), we examined the impact of the Aβ peptides on the structural parameters of the PSD, as it tracks with changes in spine morphology as a consequence of synaptic plasticity and its volume reflects the relative efficiency of the initiation of postsynaptic signaling (Harris and Stevens, 1989; Borczyk et al., 2019). Specifically, the volume, surface area, and count per region of interest (ROI) of the PSD were visualized via immunostaining of PSD95 in primary hippocampal neuronal cultures and analyzed using an automated, unbiased approach (Fig. 9; see Materials and Methods). Treatment with Aβ42 for 3 d resulted in an 18% decrease in PSD volume (mean value of 0.23 μm^3^) when compared with PSD volume (mean value of 0.28 μm^3^) of untreated cells (Fig. 9*B*). In contrast, treatment with AβCore alone and in combination with Aβ42 resulted in a rescue of PSD volume (mean value of 0.27 μm^3^) similar to that seen with untreated controls or treatment with AβCore alone (mean value of 0.26 μm^3^; Fig. 9*B*). In a similar fashion, the surface area of the postsynaptic density of Aβ42-treated cells was 13% less than untreated (mean value of 2.7 μm^2^ and 3.1 μm^2^, respectively) and treatment with the AβCore or in combination resulted in a rescue with mean values of 3.0 μm^2^ for both (Fig. 9*C*). Interestingly, there were no differences in the number of PSDs per normalized ROI volume between untreated and Aβ42. However, treatment in combination with the AβCore and Aβ42 resulted in significant increase in the number of PSDs (Fig. 9*D*). There were no statistical differences with treatment with AβCore alone.

### Comparison of impact of AβCore on presynaptic terminal volume and number

**Figure 10.**
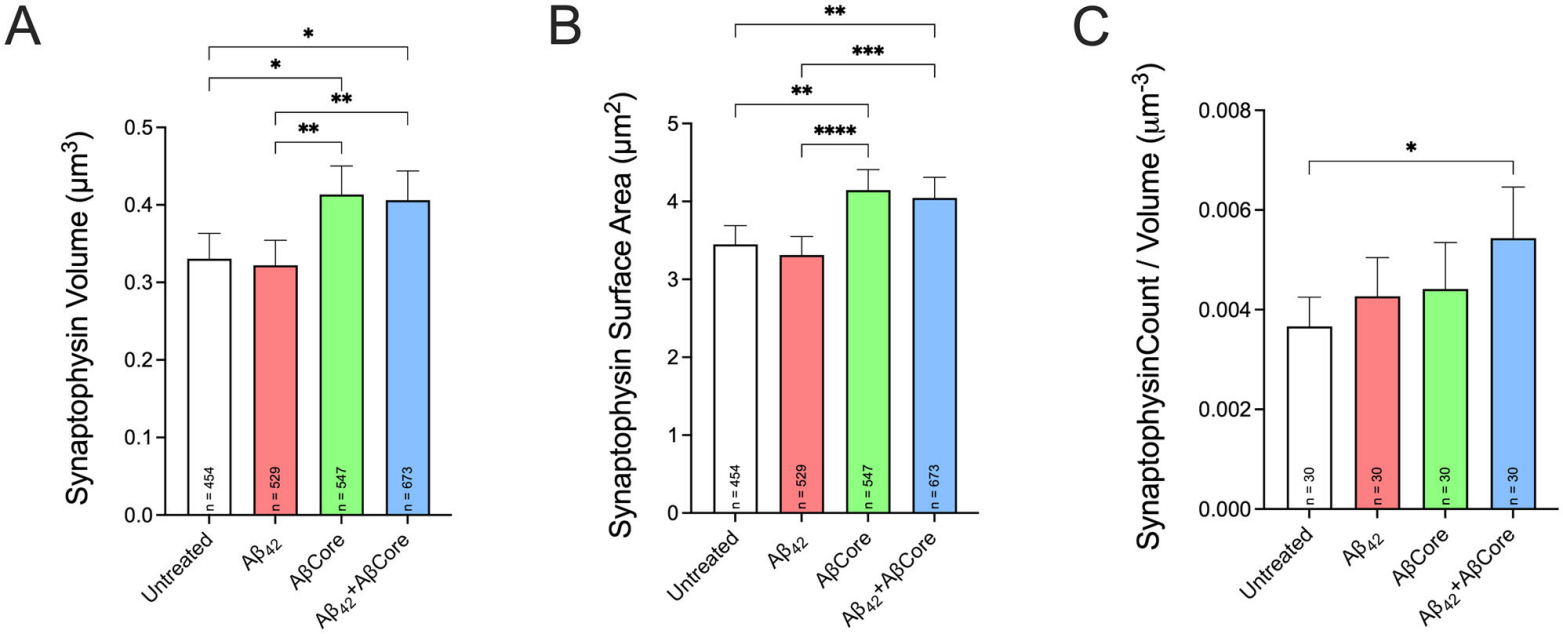
The impact of the AβCore on the structural parameters of the presynaptic terminal without or with Aβ42 treatment. Established mouse hippocampal neuron cultures were treated as described for Figure 8. 3D Leica LAS X analysis of volume (***A***), surface area (***B***), and count (***C***) of synaptophysin-positive terminals (Fig. 8) associated with the identified dendrites is expressed as mean ± 95% confidence interval (CI) with sample size (*n*). Data were analyzed by one-way ANOVA followed by Tukey’s post hoc comparisons: * < 0.05; ** < 0.01; *** < 0.001.

To investigate the impact of the AβCore on the corresponding presynaptic side of the synapse, the volume, surface area, and count per region of interest (ROI) of spine-innervating nerve terminals were assessed in primary hippocampal neuronal cultures using the synaptic vesicle marker, synaptophysin, apposed to PSDs in identified spines (Fig. 4). The volume and surface area of the presynaptic terminals in the presence of AβCore alone and in combination with Aβ42 were significantly increased when compared with the untreated condition (23–25% and 18–20%, respectively) or treatment with Aβ42 (26–28% and 22–25%, respectively; Fig. 10). While there were no statistical differences between untreated and Aβ42, the number of terminals trended toward increased counts per volume with all of the Aβ treatments when compared with untreated, with only the counts per volume significantly increased with treatment combined treatment with AβCore and Aβ42 (Fig. 10*C*).

**Figure 11.**
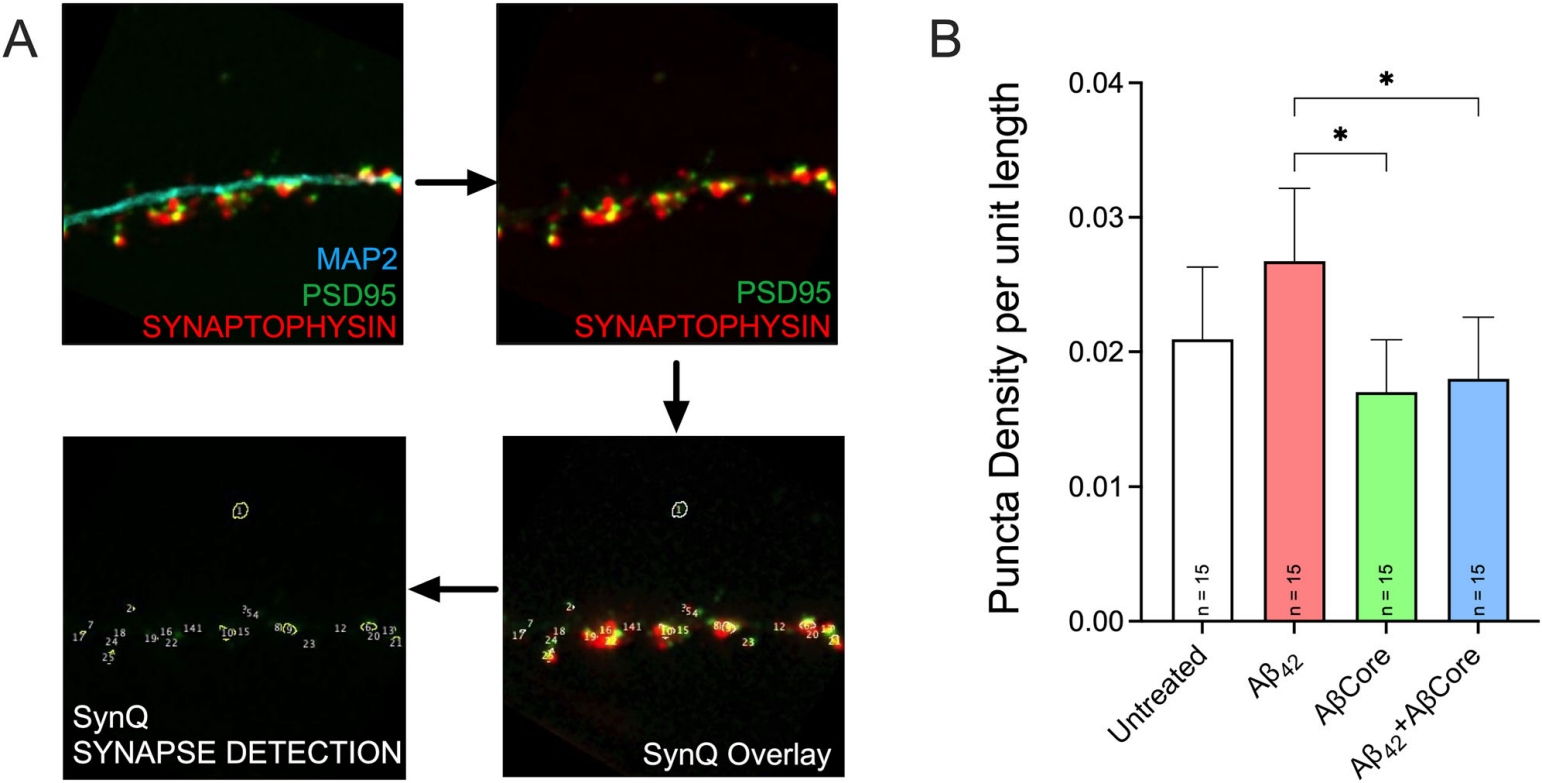
Impact of treatment with the AβCore on synapse density. Established mouse hippocampal neuron cultures were treated as described for Figure 9. ***A***, Representative images of immunocytochemistry of hippocampal cultures treated with Aβ_42_ for MAP2 (cyan), PSD95 (green), and synaptophysin (red), followed by SynQuant (SynQ) analysis of dendrite-associated PSD95 and synaptophysin as post- and presynaptic markers, respectively. Note unbiased SynQuant detection of synapses (numbered), overlaid on merged images of PSD95, and synaptophysin immunostaining (bottom panels). Arrows depict imaging processing steps. ***B***, Number of synapses as quantified by SynQuant as a puncta composite of synaptophysin and PSD95 staining. Data are expressed as mean ± 95% confidence interval (CI) with sample size (*n*). Data were analyzed by one-way ANOVA followed by Tukey’s post hoc comparisons: * < 0.05.

As an independent assessment of whole synapses, unbiased identification of synapses as puncta of apposed synaptophysin immunostaining of presynaptic terminals to PSD95 immunostaining of postsynaptic spines was achieved using the SynQuant (FIJI plugin). Interestingly, in the face of alterations of postsynaptic dendritic spines, Aβ42 treatment was associated with a trend toward altered synapse puncta density, which was normalized by cotreatment with the AβCore (Fig. 11). The AβCore alone had no significant effect on synapse puncta density compared with the untreated control.

**Figure 12.**
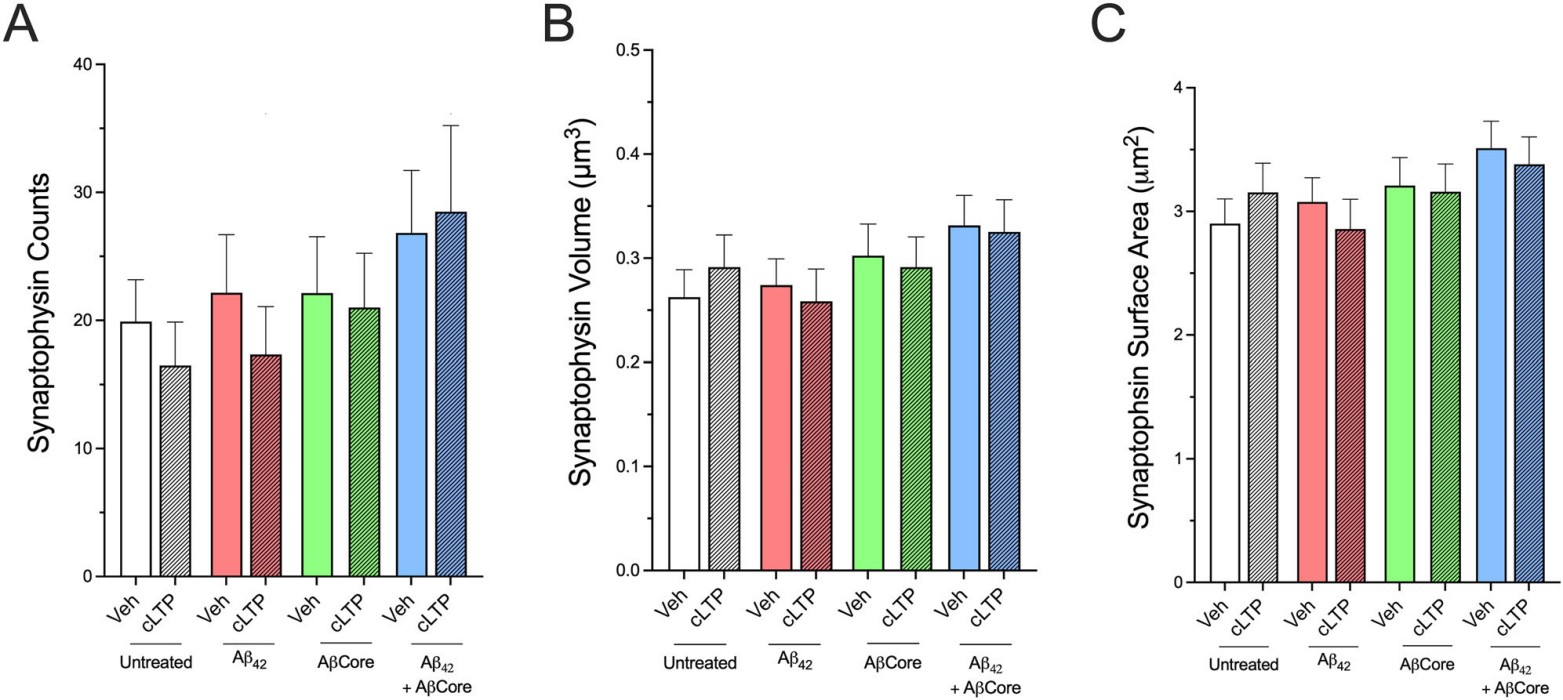
Treatment with AβCore plus Aβ_1–42_ increased presynaptic terminal volume and counts independently of cLTP. The impact of cLTP treatment on synaptic plasticity was verified by assessing GluA1 in hippocampal neuron and slice cultures, as verified in Extended Data Figure 12-1. Data are expressed as mean ± 95% confidence interval (CI) with sample size (*n*). cLTP versus vehicle analyzed by Tukey’s post hoc comparisons were not significant. *N* = 495–855.

Changes in synapses in vitro following application of Aβ42 are typically assessed in the absence of nerve stimulation, other than uncontrolled spontaneous activity. Utilizing chemical long-term potentiation (cLTP) as a controlled means to induce synaptic stimulation and plasticity (Extended Data Fig. 12-1), corresponding changes in presynaptic volume with treatment with AβCore and/or Aβ42 without or with cLTP was assessed via synaptophysin immunostaining. 3D analysis and quantification using volume, surface, and count per volume were performed as done previously. Treatment of Aβ42 showed a decreasing trend after cLTP induction (Fig. 12). cLTP treatment otherwise had no impact on the various treatments, indicating that the effects of the AβCore were independent of presynaptic activity.

10.1523/ENEURO.0044-25.2025.f12-1 Figure 12-1 **Visualization of impact of cLTP on PSDs and GluA1 expression in the postsynaptic density in primary hippocampal cultures and GluA1 expression in OHSCs. *A***, Representative images of GluA1 (yellow), PSD95 (green), synaptophysin (red) and MAP2 (cyan) immunostaining of primary hippocampal neuron cultures subjected to cLTP or not (vehicle) in a 3D rendering via Leica LAS X. A separate image of synaptophysin and PSD95 staining overlay only shows apposition of pre- and postsynaptic components separate from the expanded GluA1 staining with cLTP. ***B***, Impact of cLTP on GluA1 immunostaining (red) of organotypic hippocampal slice cultures (OHSC). Representative images of GluA1 expression confirm increased GluA1 immunostaining with cLTP-induced synaptic plasticity. Download Figure 12-1, TIF file.

## Discussion

In Alzheimer's disease functional compromise and corresponding morphological alterations in postsynaptic dendritic spines precede eventual synapse loss in select areas of the brain, leading to memory and cognitive deficits (reviewed in Penzes et al., 2011). We have previously shown the protective rescue by the AβCore against full length Aβ_42_-induced: neurotoxicity (oxidative stress; apoptosis), neuronal Ca^2+^ hyperexcitation, altered synaptic signaling, deficits in synaptic plasticity, and gliotoxicity (Lawrence et al., 2014; Forest et al., 2018, 2021; Roberts et al., 2021, 2024; Lantz et al., 2023). However, the potential of the AβCore to protect against Aβ-associated structural pathology of dendritic spines (morphology and density) has not been examined. Dendritic spines are highly specialized structures found at excitatory synapses, whose size and intracellular signaling in response to varying patterns of presynaptic input regulate synapse efficacy and plasticity. In AD models, decreased spine density (number of spines per unit length of dendrite) and altered spine size (spine head volume) manifested as varying changes in the types of spines have been widely reported (Calabrese et al., 2007; Bączyńska et al., 2021).

Our results examining the impact of Aβ treatment of cultured primary hippocampal neurons using different approaches to spine visualization revealed a dramatic and relatively rapid change in dendritic spine size across the whole of the spine population, with a more delayed impact on spine number. The resulting reduction in spine cross-sectional area indicated an Aβ-induced reduction in spine volume, and hence shrinkage, consistent with previously noted findings both for AD pathology models and AD. Interestingly, there was no differential change in putative subtypes of spines, such as mushroom-like, as the spine distribution was continuous across the population, consistent with previous detailed observations of spine ultrastructure (Arellano et al., 2007). Confirmation of altered postsynaptic structure with Aβ treatment, which correlates with synaptic efficacy, was obtained via volumetric assessment of the PSDs in spines using 3D analysis. Similar changes in PSD surface area with Aβ treatment were observed in parallel. Corresponding changes for presynaptic terminals to Aβ treatment were not observed over the observed time frame. Interestingly, Aβ increased actual synapse number, perhaps as an early compensatory mechanism (Zhou et al., 2022), which later converts to synaptic terminal loss largely via glia-mediated synaptic pruning. Moreover, these changes occurred independently of synaptic activity, further suggesting that Aβ acts directly to compromise the synaptic components. While the time frame for direct impact of Aβ in vitro on isolated, cultured neurons is greatly accelerated as compared with in vivo in AD pathology models or AD, the variation in local Aβ levels and other protective mechanisms (e.g., BDNF; Arancibia et al., 2008) act to prolong the time course for synaptotoxicity in vivo. Nonetheless, the results in the present report support other findings in which altered synaptic morphology precedes synapse loss.

For all Aβ-induced changes, cotreatment with the neuroprotective AβCore normalized presynaptic and postsynaptic structure and density across the synaptic population. There was also an apparent impact of the AβCore alone to increase spine density and the PSD count. These latter results support the notion that the AβCore likely both interferes with the action of Aβ42 at cell surface receptors and differentially activates intracellular signaling (Forest et al., 2018, 2021). How such direct actions of the AβCore appear to increase spine density remains to be determined. One possibility related to the AβCore-associated normalization of spine size is structural stabilization leading to reduced spine turnover, which could be assessed via repeated imaging of the same spines over hours to days. Furthermore, use of single-synapse (Phongpreecha et al., 2021) and single-spine (Bosch et al., 2014) molecular analyses coupled with spine size sorting to characterize intracellular pathways engaged by Aβ42 and those differentially regulated by the AβCore in the context of structural changes in dendritic spines and spine cytoskeleton would be useful for understanding the broader protective mechanisms for the AβCore and the identification of novel targets.

Structural changes in spine size are linked to changes in cytoskeletal dynamics (Matus, 2000; Johnson and Ouimet, 2006), being evident in the present study in the Aβ-induced reduction in the relative expression in spines of the cytoskeletal protein drebrin, primarily used here as a dendritic spine marker. It would therefore be predicted that the normalization of drebrin expression by cotreatment with AβCore is reflective of normalization of the actin cytoskeleton. Future studies could examine more directly the acute regulation by the AβCore of actin dynamics, spine size, and spine turnover at individual synapses using time-lapse imaging and the impact of the AβCore on these structural parameters in AD models over long-term treatment. Of particular interest in regard to the molecular mechanisms for spine structural changes will be analyses of actin dynamics regulators in addition to drebrin, such as the aforementioned CaM kinase II, calcineurin, α-actinin, myosins V and VI, Arp2/3 and Rac1, cofilin and LIMK, and MARCKS and PI(4,5)P_2_ (Knobloch and Mansuy, 2008; Penzes et al., 2011; Soria Fregozo and Pérez Vega, 2012; Chazeau and Giannone, 2016; Borovac et al., 2018; Okabe, 2020; Pelucchi et al., 2020; Calabrese and Halpain, 2024). The impact of the AβCore in AD pathology models on spine postsynaptic neurotransmitter receptor expression and regulation (e.g., AMPA-type glutamate receptors), BDNF signaling, filopodia, and tau pathology in the context of the presence of ApoE variants will also be important to investigate.

### Conclusion

The normalization of spine size and density on cotreatment of the AβCore with Aβ suggests that pretreatment with AβCore could be used for protection against Aβ-induced spine loss in AD pathology.

## Synthesis

Reviewing Editor: Timothy Jarome, Virginia Polytechnic Institute and State University Decisions are customarily a result of the Reviewing Editor and the peer reviewers coming together and discussing their recommendations until a consensus is reached. When revisions are invited, a fact-based synthesis statement explaining their decision and outlining what is needed to prepare a revision will be listed below. The following reviewer(s) agreed to reveal their identity: Andrew Arrant.

Overall the manuscript is well done. The reviewers have a few comments that need to be addressed though:

1. This in vitro work follows the extensive in vivo work on core peptide, unlike most other work where in vitro work follows in vivo work. Authors have not covered the latest literature for novel methods of synaptic evaluation introduction, result or discussion ( PMID: 34910503, 30465796, 35099792). Authors can discuss this and similar other technologies in the context of their results and perspective.

2. Report rpm as rcf or g. As rpm differs between rotors on the same machine and or between centrifugal devices, whereas rcf/g are universal.

3. There is always a concern about such in-depth approaches for reproducibility. Encourage adding more granular detail for the cell culture and method for synaptic analysis.

4. Fig S3: how can PSD95 (post-synaptic) and Synaptophysin(pre-synaptic) markers present in separate places, have nearly 100% overlay, especially in the cLTP panel? Additionally, it would be great to include more discussion on why AB core might be increasing the number of PSD-95 and synaptophysin-related puncta.

5. It is not clear how the distribution data in Figs. 5, 6, and 8 were analyzed. The methods section mentions regression analysis, but further detail is needed to on the type of analysis. These data seem appropriate for Kolmogorov-Smirnov test. Additionally, statistical significance between groups should be indicated on the figure.

6. Since chemical LTP did not alter any spine parameters, it is not clear what these data add to the manuscript. Given these negative results, it is also not clear if the chemical LTP protocol was effective. Inclusion of some evidence of plasticity would strengthen these results.

## Author Response

Response to eNeuro eN-NWR-0044-25 Review 2-13-25 "Overall the manuscript is well done. The reviewers have a few comments that need to be addressed though":

1. This in vitro work follows the extensive in vivo work on core peptide, unlike most other work where in vitro work follows in vivo work. Authors have not covered the latest literature for novel methods of synaptic evaluation introduction, result or discussion (PMID: 34910503, 30465796, 35099792). Authors can discuss this and similar other technologies in the context of their results and perspective.

We appreciate the suggestion to expand our discussion of synaptic evaluation. We have incorporated references to approaches and molecular targets for individual synaptic evaluation in the Discussion, especially where they can complement and/or extend our findings.

2. Report rpm as rcf or g. As rpm differs between rotors on the same machine and or between centrifugal devices, whereas rcf/g are universal.

This is an important technical point. We have made the change from rpm to g.

3. There is always a concern about such in-depth approaches for reproducibility. Encourage adding more granular detail for the cell culture and method for synaptic analysis.

We share the need for adequate detail for assessment of reproducibility. We have added information about assessing cell culture viability and reproducibility and synaptic analysis (Methods).

4. Fig. S3 (Extended Data Fig. 12-1): how can PSD95 (post-synaptic) and Synaptophysin (pre-synaptic) markers present in separate places, have nearly 100% overlay, especially in the cLTP panel? Additionally, it would be great to include more discussion on why AB core might be increasing the number of PSD-95 and synaptophysin-related puncta.

On re-examination of the images, we agree that the overlap of staining in the cLTP- treated cultures (Fig. S3, lower sequence) is somewhat problematic, not seen in the vehicle control cultures (Fig. S3, upper sequence). To some extent the more expansive GluA1 staining (which extends across the whole spine, unlike the PSD) obscured the apposition of synaptophysin to PSD95, as noted by some of the synaptophysin (red) evident in the lower merged image. We have added an overlay of synaptophysin and PSD95 only to demonstrate their apposition. To address the point of this experiment, we have added in an image series of cLTP-induced changes in GluRs in hippocampal slice cultures (see also point 6), showing the impact of the cLTP treatment on an ex vivo preparation preserving network circuitry.

We have added more discussion regarding the AbetaCore-associated increase in spine density. The AbetaCore had no significant effect on presynaptic terminal density or synaptic puncta density.

5. It is not clear how the distribution data in Figs. 5, 6, and 8 were analyzed. The methods section mentions regression analysis, but further detail is needed to on the type of analysis. These data seem appropriate for Kolmogorov-Smirnov test. Additionally, statistical significance between groups should be indicated on the figure.

The regression analysis was used solely to assess slope values in the linear portion of the fractional distribution curves. This has been clarified in the Statistics section. As noted in the Results (p16), the slope values provide quantification of overall size changes across the spine population.

Kolmogorov-Smirnov tests were performed prior to parametric statistical analysis to assess normality (Gaussian) of data distribution.

Adding statistical analyses to the plots of fractional distributions is problematic. Dunn's pairwise post hoc test for Kruskal-Wallis statistical analyses for Fig. 5B&C has been incorporated into the text. This non-parametric test is most appropriate for comparison of median values derived from the ranked data, here fractional distribution. Statistical analyses of the data presented in Figs. 6 and 8 are provided in accompanying tables.

6. Since chemical LTP did not alter any spine parameters, it is not clear what these data add to the manuscript. Given these negative results, it is also not clear if the chemical LTP protocol was effective. Inclusion of some evidence of plasticity would strengthen these results.

We have added an image sequence to Fig. S3 (Extended Data Fig. 12-1B) of immunostaining of vehicle vs. cLTP-treated hippocampal slice cultures showing the impact on expression of GluRs where the inherent circuitry is intact. These data provide evidence of the efficacy of cLTP to induce plasticity, as indicated by the upregulation of AMPA-type glutamate receptors.
