# Supplementary figures and images for: The Beta Amyloid Core Hexapeptide Protects against Full-Length Beta Amyloid-Induced Alteration of Dendritic Spine Morphology and Density
Source: eNeuro. 2025 Sep 9;12(9):ENEURO.0044-25.2025. doi: 10.1523/ENEURO.0044-25.2025 (PMC12439754; doi:10.1523/ENEURO.0044-25.2025)

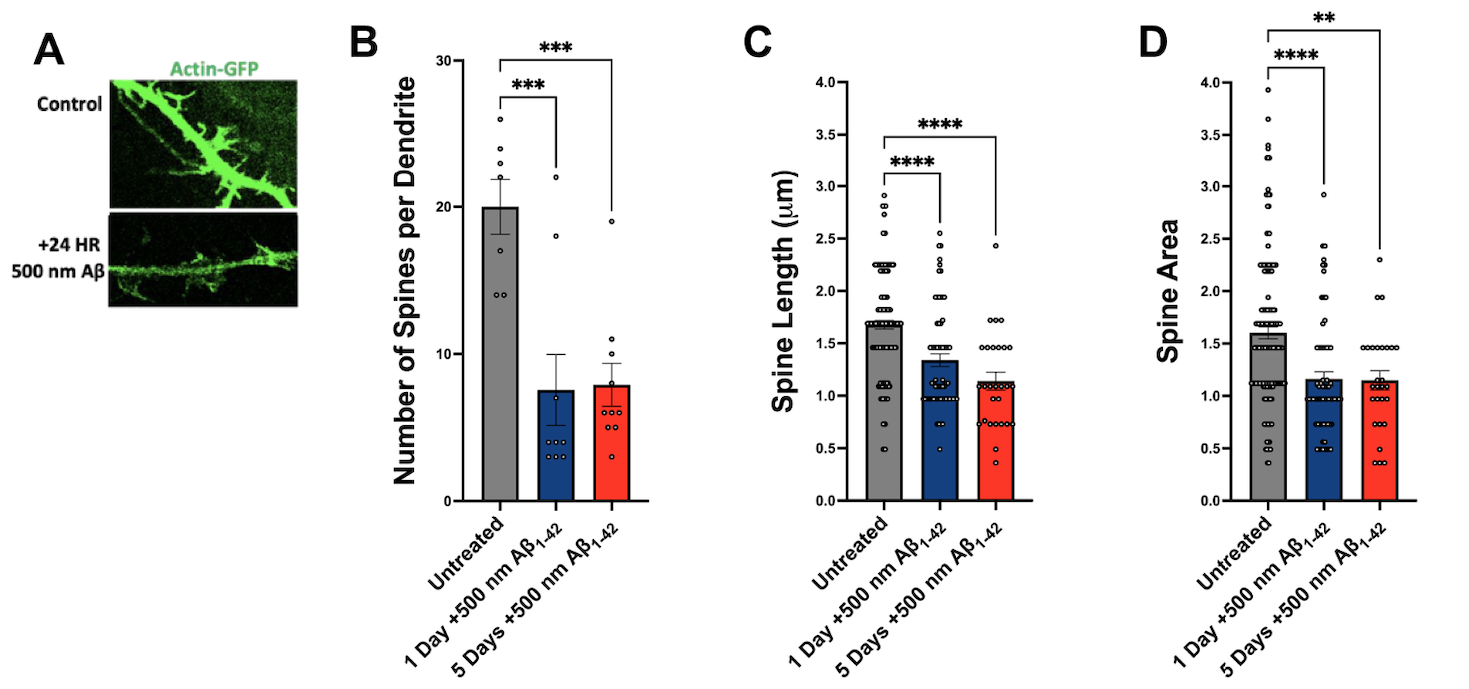

Supplement: Figure 5-1 — Aβ42-induced alterations in dendrite spine parameters of hippocampal neurons visualized with Actin-GFP. A, Representative dendrites of neurons labeled with Actin-GFP using BacMam transduction, following treatment or not with Aβ42 for 1 or 5 days. Graphs are plots of summarized analyses of the number of spines per unit length of dendrite (B), spine length: μm (C) and spine area: μm2 (D). Download Figure 5-1, TIF file. [file eneuro-12-ENEURO.0044-25.2025-s002.tif]

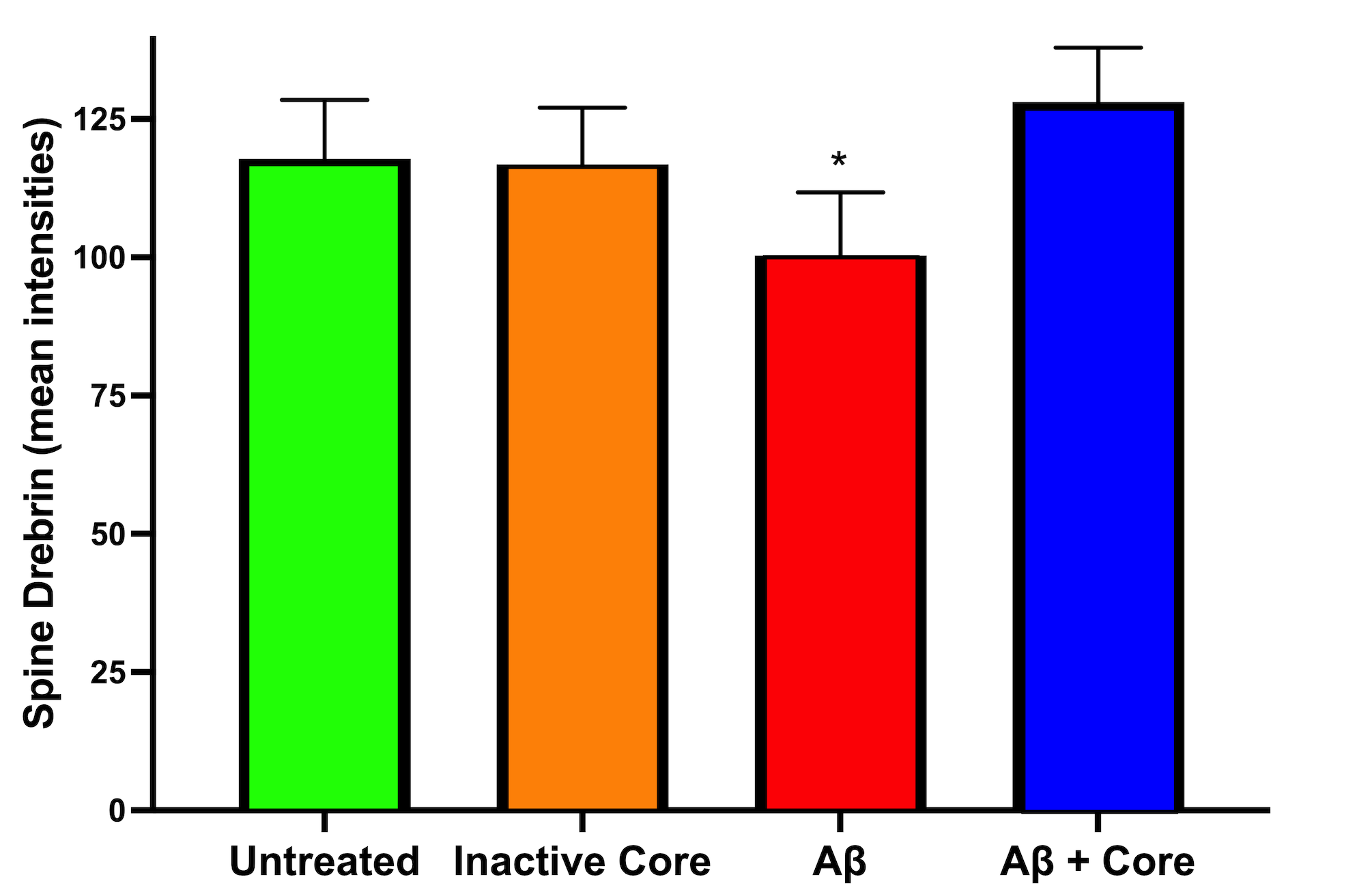

Supplement: Figure 5-2 — Impact of AβCore on Aβ42-induced changes in Drebrin expression. Rescue of Aβ-induced reduction of drebrin immunocytochemical expression (mean integrated fluorescent intensity values in arbitrary units) in identified dendritic spines by co-treatment with the AβCore (Core). Data are means +/- SD; Sample sizes (# of spines analyzed): 551 Untreated; 543 Inactive Core; 324 Aβ; 405 Aβ + Core. *p=0.03 Aβ vs. Untreated by post hoc comparison following ANOVA. Download Figure 5-2, TIF file. [file eneuro-12-ENEURO.0044-25.2025-s003.tif]

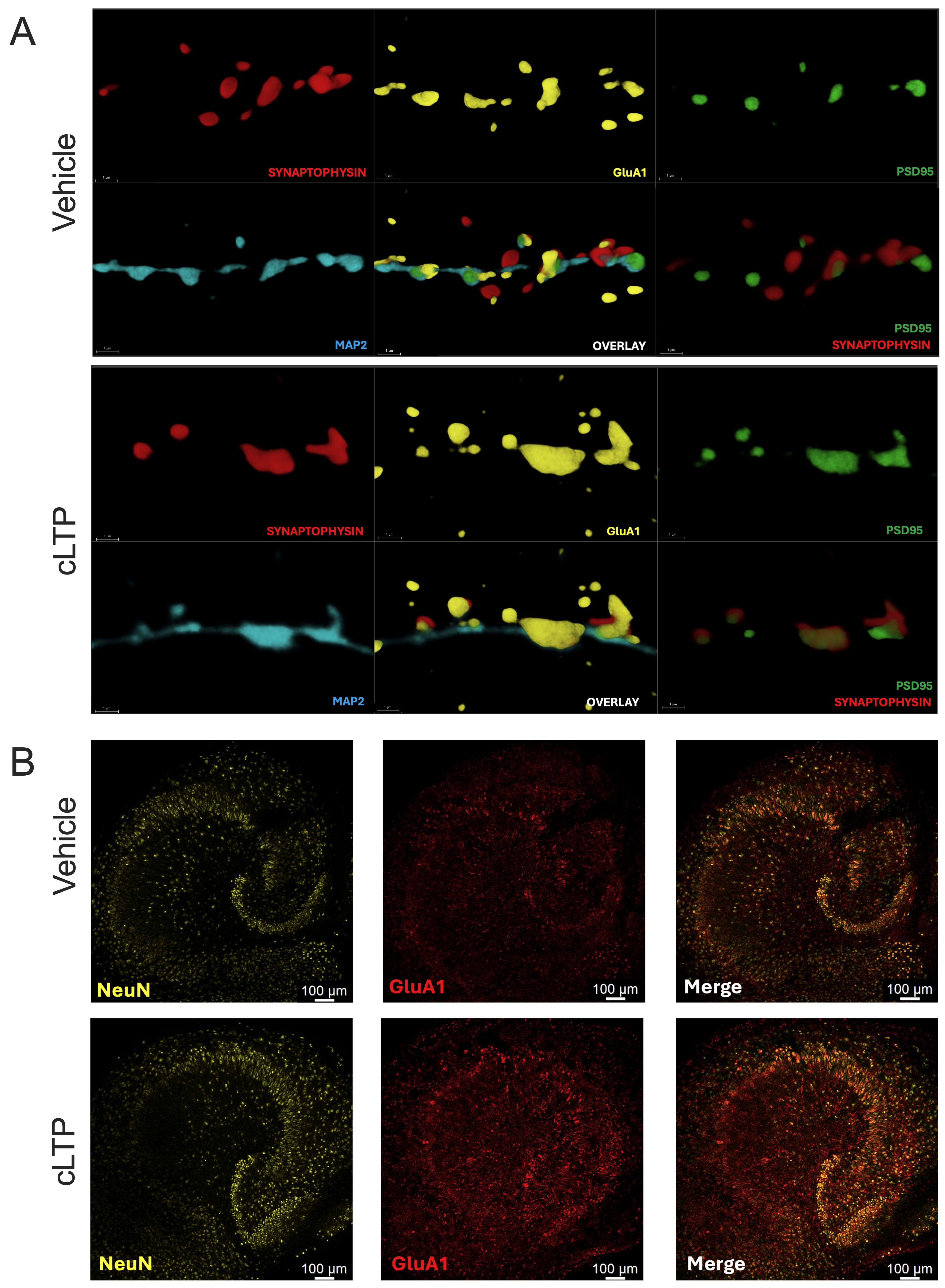

Supplement: Figure 12-1 — Visualization of impact of cLTP on PSDs and GluA1 expression in the postsynaptic density in primary hippocampal cultures and GluA1 expression in OHSCs. A, Representative images of GluA1 (yellow), PSD95 (green), synaptophysin (red) and MAP2 (cyan) immunostaining of primary hippocampal neuron cultures subjected to cLTP or not (vehicle) in a 3D rendering via Leica LAS X. A separate image of synaptophysin and PSD95 staining overlay only shows apposition of pre- and postsynaptic components separate from the expanded GluA1 staining with cLTP. B, Impact of cLTP on GluA1 immunostaining (red) of organotypic hippocampal slice cultures (OHSC). Representative images of GluA1 expression confirm increased GluA1 immunostaining with cLTP-induced synaptic plasticity. Download Figure 12-1, TIF file. [file eneuro-12-ENEURO.0044-25.2025-s004.tif]
